# Albumin does not induce IL-6 release and toll-like receptor activation in vitro: Role of endotoxin contamination and biochemical modifications

**DOI:** 10.1016/j.bbadva.2025.100174

**Published:** 2025-11-17

**Authors:** Margret Paar, Vera H. Fengler, Martina Schweiger, Christine Rossmann, Christoph Nusshold, Martina Mairold, Doris Payerl, Gerhard Cvirn, Karl Oettl

**Affiliations:** a Division of Medicinal Chemistry, Otto Loewi Research Center, Medical University of Graz, Neue Stiftingtalstraße 6, 8010 Graz, Austria; b Institute of Molecular Biosciences, University of Graz, Humboldtstraße 50, Heinrichstraße 31, 8010 Graz, Austria

**Keywords:** Human serum albumin, Albumin redox state, Glycated albumin, Endotoxin contamination, Cytokine release, Toll-like receptors, Inflammation

## Abstract

Human serum albumin (HSA) is widely used in both clinical and research settings, yet its potential to stimulate immune responses remains poorly understood. In this study, we systematically investigated various therapeutic, recombinant and plasma-derived albumin preparations for their ability to induce pro-inflammatory cytokine release by human peripheral blood mononuclear cells (PBMC) and activate Toll-like receptors (TLR) in HEK-Blue™ TLR4 and TLR2 reporter cell lines. All albumin preparations were characterized in terms of their redox state of cysteine-34, glycation level, non-esterified fatty acid content, and endotoxin contamination Therapeutic albumin, as may be expected, did not induce IL-6-release nor TLR activation. However, several commercial research grade albumins, including fatty acid-free, glycated, and recombinant preparations, significantly induced IL-6 release and activated TLR4 and TLR2 signaling. These inflammatory effects were strongly correlated with the endotoxin content, while no consistent associations were found with albumin redox state, degree of glycation, or fatty acid load. Albumins with more reduced cysteine-34 content showed lower pro-inflammatory activity, but this correlation was lost when controlling for endotoxin levels, suggesting confounding by endotoxin contamination. Direct testing of isolated albumin redox fractions and in vitro glycated albumins confirmed that neither oxidation nor glycation of albumin, in the absence of endotoxin, triggered inflammatory responses in PBMCs. Inducing effects were found to be related to contaminating ligands and not to albumin itself. Our findings emphasize the need for rigorous product characterization and highlight the critical importance of endotoxin as a confounding factor in immune assays involving research grade albumin preparations.

## Highlights

• Research grade albumin may stimulate IL-6 release by PBMC via activation of TLR2/4.
• IL-6-release and TLR-activation correlate with endotoxin contaminations.
• Albumin redox state, glycation and fatty acids have no impact on IL-6-release.
• Albumin needs rigorous characterization prior to usage in cell culture experiments.
• Therapeutic albumin did not show immune-stimulatory effects.

## 1 Introduction

Human serum albumin (HSA), the most abundant protein in plasma, plays a central role in maintaining oncotic pressure, transporting ligands, and is involved in redox homeostasis. Beyond its classical functions, emerging evidence suggests that albumin may influence immune responses, also via its redox state. Albumin’s redox function is primarily mediated by the thiol group of cysteine-34 (Cys-34), albumin’s sole free cysteine residue not involved in intramolecular disulfide bonding. Based on the redox state of Cys-34 three distinct fractions of albumin can be distinguished: human mercaptalbumin (HMA), the reduced form retaining a free thiol group; human non-mercaptalbumin 1 (HNA1), harboring a reversible disulfide bond with a low-molecular-weight thiol such as cysteine; and human non-mercaptalbumin 2 (HNA2), representing an irreversibly oxidized form in which Cys-34 is converted to sulfinic or sulfonic acid, typically through exposure to strong oxidants such as hydrogen peroxide. HNA1 has been reported to exhibit immune stimulatory effects in peripheral blood mononuclear cells (PBMC) likely through the activation of pattern recognition receptors (PRR) such as toll-like receptors (TLR) [1].

Besides oxidation of Cys-34, non-enzymatic glycation of e.g., lysine residues, is another common post translational modification (PTM) of albumin with physiological relevance as it reflects blood glucose levels over the past 2 to 3 weeks [2]. Elevated levels of glycated albumin are an indicator for hyperglycemia and are associated with the presence of diabetic retinopathy, nephropathy, and cardiovascular complications in diabetic patients [[3], [4], [5], [6]]. Furthermore, glycated albumin appears to have pro-inflammatory effects [7].

Despite these findings, the precise mechanisms how albumin modifications induce immune activation remain poorly characterized, which is particularly of high importance in the context of commercially available albumin preparations, which are widely used in both research and clinical settings. To address this gap, we systematically analyzed several commercial albumin products as well as albumins purified from plasma of donors for their potential to induce cytokine release by PBMC and to activate TLR4- and TLR2-mediated signaling. Furthermore, we characterized these albumins with respect to their redox state, fatty acid (FA) content, and degree of glycation—factors that all may influence the immune stimulatory activity of albumin [[8], [9], [10]].

An important confounding factor in the assessment of inflammatory effects is the presence of endotoxin (lipopolysaccharide, LPS), a component of the outer membrane of Gram-negative bacteria and potent TLR4 agonist commonly found as a contaminant in protein preparations. Notably, our analyses revealed that some commercial albumin products contained substantial levels of endotoxin. However, standard limulus amebocyte lysate (LAL)-based endotoxin detection methods often underestimate endotoxin levels when it is masked by protein, a phenomenon known to occur with albumin and other serum proteins [11]. This complicates the interpretation of immunological data and underlines the need for careful endotoxin control and more robust quantification methods for scientific preparations, and not only for therapeutic albumin solutions which are subjects to strict standards and of which applications are safe.

Within the present study, we aimed to disentangle the effects of albumin's redox state from those of protein-bound endotoxin and/or other albumin-associated molecular features in inducing inflammation. For this purpose, we investigated the release of the pro-inflammatory cytokine IL-6 by PBMC and the activation of TLR4 and TLR2 in HEK-Blue™ TLR reporter cell lines treated with or without the different aforementioned albumins. Our findings highlight the complexity of albumin-mediated immune modulation and point towards critical quality criteria that must be considered when interpreting immune responses in vitro using albumin-containing formulations.

## 2 Materials and methods

### 2.1 Commercial albumin products

Albumin from human serum (A1653, Lot SLBX4252), fatty acid free albumin (A1887 Lot SLCL2530 and SLBM7779V), fatty acid free and globulin free albumin (A3782 Lot SLCH3002), glycated albumin (A8301 Lot SLCG2829), and recombinant albumin (from rice) (A9731 Lot SLCJ1701) were obtained from Sigma Aldrich. oHSA (recombinant albumin from rice) was obtained from Oryzogen (Wuhan, China). Therapeutic human albumin was obtained as a 20 % solution (Behring). Commercial albumin products were opened exclusively under sterile conditions in a laminar flow hood and collected with sterilized spatulae. The powders were dissolved in endotoxin free water, subsequently sterile filtered and stored at −80 °C until use. In case of therapeutic albumin, the stabilizer N-acetyl-D,L-tryptophane was removed by size-exclusion chromatography using a PD-10 desalting column (GE Healthcare), eluted in endotoxin free water, sterile filtered and also stored at -80 °C. One aliquot was used for albumin quantification using the bromocresol green (BCG) method.

### 2.2 Preparation of albumin from healthy donors

For plasma preparation of HSA and the isolation of peripheral blood mononuclear cells, ethylene diamine tetra acetic acid (EDTA) blood was drawn from healthy donors. Blood samples were collected at the Division of Medicinal Chemistry as approved by the Ethics Committee of the Medical University of Graz (29–460 ex 16/17 and 35–166 ex 22/23). All volunteers gave their informed consent prior to collection of samples. Blood samples were centrifuged immediately at 4 °C to separate plasma. Plasma of four donors each was pooled and the albumin prepared by loading it to a HiTrap™ Blue HP column (GE Healthcare, Solingen, Germany). After equilibrating the column with 50 mM potassium dihydrogenphosphate (pH 7.0), albumin was eluted with 50 mM potassium dihydrogenphosphate solution containing 1.5 M potassium chloride (pH 7.0). Pooled fractions were applied to a PD-10 desalting column, eluted in endotoxin free water, sterile filtered and also stored at -80 °C. The resulting albumin preparations were termed as C1 and C2.

### 2.3 In vitro reduction/oxidation of albumin

For the preparation of reduced (HMA) and oxidized (HNA1 and HNA2) albumin fractions, EDTA blood of three healthy donors was separately incubated in the presence of 1 mg/mL N-acetylcysteine for 1 h at room temperature (RT). This leads to a reduction of HNA1, resulting in mainly HMA. HMA-enriched plasma was then obtained upon centrifugation, and HMA prepared as described above for albumin. In parallel, albumin of these donors was also prepared from plasma without treatment, the resulting control albumin is termed C3. To obtain HNA1, the purified HMA was incubated with 17 mmol/L cystine at 37 °C for at least 24 h. HNA2 was prepared by incubating HMA with 45 mmol/L hydrogen peroxide (H_2_O_2_) for 1 h at RT. Complete reduction/oxidation was examined by high-performance liquid chromatography (HPLC) analysis and residual cystine or H_2_O_2_ removed by dialysis against distilled water at 4 °C. All fractions were subsequently sterile filtered and stored at −80 °C until further usage.

### 2.4 In vitro glycation of albumin

Research grade albumin as well as isolated albumin from pooled plasma of four healthy control donors (C2) were buffered in PBS (pH 7.4), sterile filtered, and diluted to a concentration of 40 mg/mL. The solutions were then incubated, in triplicates, in the absence (0 mmol/L) or presence of 25 mmol/L or 100 mmol/L, respectively, of glucose solution (GibcoTM by Life Technologies, Thermo Fisher Scientific) for 3 weeks at 37 °C. Subsequently, albumin solutions were re-buffered by five repeated steps of concentrating via Amicon Ultra-0.5 centrifugal filter units (cutoff 30 kDa; Millipore, Merck) followed by dilution with PBS. The final albumin concentration was determined by bromocresol method using Albumin liquicolor (Human Diagnostics Worldwide, Wiesbaden, Germany). Fructosamine content was determined by Glycated Serum Protein Assay (Diazyme Europe GmbH, Dresden, Germany) and normalized to the respective albumin concentration (GSP/ALB).

### 2.5 Complexation of HSA and fatty acids using lipolysis-stimulated murine fat pads

Gonadal adipose tissue of C57BL/6 mice was excised and washed in RPMI (without additives) containing 100 IU/mL heparin. Fat pads were incubated in 48 well plates. 400 µL of the respective medium containing three different donor albumins (C3) (5 mg/mL each) and 10 µM forskolin were incubated for 90 min. Thereafter the medium was collected and 15 µL medium were analyzed for FA content using a commercial kit (FUJIFILM Wako Chemicals Europe GmbH, Neuss, Germany). As a background control, all prepared media (containing human albumin) were pooled. The remaining media containing the respective fatty acid-loaded albumin fractions were stored at −80 °C. Fat pads were lyzed in 500 µL lysis solution (0.1 % SDS, 0.3 mol/L NaOH) for 2 h at 70 °C, the fat fraction was removed by centrifugation (10 min, high speed), and protein content was determined using BCA reagent (Pierce, Rockford, IL) and BSA as standard.

### 2.6 HPLC analysis of albumin redox state

To determine its redox state albumin was split into its different fractions (HMA, HNA1, and HNA2) by HPLC as described by Hayashi et al. [12]. Briefly, samples were diluted with 0.1 M sodium phosphate, 0.3 M sodium chloride solution (pH 6.87) and filtered through Whatman 0.45 µm nylon filters (Bartelt Labor- & Datentechnik, Graz, Austria). 20 µL of the diluted samples were injected into the HPLC system. Separation was performed using a Shodex Asahipak ES-502 N 7C anion exchange column with 50 mM sodium acetate, 400 mM sodium sulfate solution (pH 4.85) as mobile phase. Gradient elution was performed with 0 to 6 % ethanol at a flow rate of 1 mL/min (described in detail in [13]). The column was kept at 35 °C. Detection was carried out by fluorescence at 280/340 nm and quantification was based on the areas of the individual peaks as determined using Peak Fit software (Version 4.12, SPSS Science, Chicago, IL, USA) by fitting Gaussian curves to the chromatograms.

### 2.7 Biochemical analyses

The non-esterified fatty acid content of the respective samples was determined using a commercial kit (FUJIFILM Wako Chemicals Europe GmbH, Neuss, Germany) and normalized to the protein content, which was determined using BCA reagent (Pierce, Rockford, IL). Albumin concentration was measured with Albumin (BCG) Assay Kit (Abcam).

### 2.8 HEK-Blue™ TLR cell lines

HEK-Blue™ TLR2 and TLR4 cells were cultured in 75 cm^2^ flasks containing Dulbecco's modified Eagle medium (DMEM) supplemented with 4.5 g/L glucose, 10 % (v/v) FBS, 50 µg/mL penicillin, 50 µg/mL streptomycin, 100 µg/mL Normocin™, 2 mM l-glutamine, and 1x HEK-Blue™ Selection (a mixture containing several selective antibiotics, that guarantee the persistent expression of transgenes introduced to HEK-Blue cells) at 37 °C and 5 % CO_2_. Cells were passaged before reaching 100 % confluency and only passages below 25 were used for experiments.

### 2.9 HEK-Blue™ TLR2/4 stimulation with potential TLR agonists

HEK-Blue™ TLR2 or TLR4 cells were plated in 48 well plates (10^5^ cells/well) and allowed to settle for 9 h at 37 °C before experimental start. Experiments were carried out in HEK-Blue™ Detection medium by incubating the cells in the absence (H_2_O bidest. served as vehicle control) or presence of various albumins (1 mg/mL, final concentration), respectively, for at least 18 h at 37 °C. Treatment of HEK-Blue™ cells with selective agonists for TLR4 (LPS; 10 ng/mL final concentration) or TLR2 (lipoteichoic acid (LTA); 20 ng/mL final concentration) served as positive controls for selective TLR activation. In some experiments the specific TLR4 inhibitor CLI-095 was present during cell treatment at concentrations of 3 µmol/L (final concentration). All incubations were performed in duplicates. 100 µL of the culture medium were transferred to 96 well plates and secreted embryonic alkaline phosphatase (SEAP) activity was assessed spectrophotometrically by measuring the absorbance at 640 nm using a microplate reader. Results are expressed as % of control (vehicle) and represent mean + SD of at least two independent incubations.

### 2.10 Isolation and culture of human peripheral blood mononuclear cells (PBMC)

PBMC were isolated from a maximum of 45 mL EDTA blood collected from healthy donors by density gradient centrifugation. In brief, blood was centrifuged at 1000 rpm for 10 min at RT. Plasma was removed and replaced by the same volume of PBS pH 7.4. Blood was then loaded on Ficoll-Paque Plus (GE Healthcare, Chicago, Illinois, USA) and centrifuged at 1580 rpm for 30 min at RT. PBMC were harvested and incubated in ACK-lysis buffer (150 mM NH_4_Cl, 10 mM KHCO_3_, 0.1 mM Na_2_-EDTA; pH 7.3) for 10 min. After centrifugation at 1400 rpm for 10 min at RT, the cell pellet was washed in 10 mL PBS and again centrifuged. Isolated PBMC were suspended in serum-free RPMI 1640 medium (GibcoTM) containing antibiotics (100 U/mL penicillin and 100 µg/mL streptomycin) and seeded at a density of 6 × 10^5^ cells per 48-well. PBMC were incubated in 0.4 mL of RPMI 1640 medium in the presence of 1 mg/mL of albumin preparations at 37 °C and 5 % CO_2_ for 4 h. Supernatants were subjected to ELISA to determine IL-6 concentrations.

### 2.11 Enzyme-linked immunosorbent assay (ELISA)

IL-6 release to the culture medium of PBMC incubated in the absence or presence of albumins was determined by Human IL-6 ELISA (ImmunoTools, Friesoythe, Germany) according to the manufacturer’s protocols. Samples were analyzed either undiluted or following dilution (up to 1:50) in RPMI medium to ensure accurate quantification within the standard curve range.

### 2.12 Quantification of the endotoxin content

To determine the protein-masked endotoxin content, 2 mg/mL of each albumin preparation were incubated with Proteinase K (0.1 mg/mL; Sigma Aldrich) for 18 h at 55 °C and gentle shaking. Subsequently, Proteinase K was heat-inactivated for 20 min at 75 °C. Completeness of digestion was confirmed by native SDS Page followed by Coomassie Brilliant Blue staining and destaining with 10 % acetic acid. Digested samples were diluted 1:2 with sterile PBS and endotoxin content was determined using PierceTM Chromogenic Endotoxin Quant Kit (Thermo Fisher Scientific) according to the manufacturers protocol. As High Standards (0.1–1.0 EU/mL; 0.1 EU equals 20 pg endotoxin) were used, standards and samples were incubated with Amebocyte Lysate for 12 min at 37 °C followed by the incubation with Chromogenic Substrate Solution for 6 min. To stop the reaction, 25 % acetic acid was added and the OD measured at 405 nm immediately after assay completion.

### 2.13 Statistical analysis

Data were stored in a Microsoft Excel sheet and GraphPad Prism Version 10 (GraphPad Software, San Diego, CA, USA) was used for statistical analyses and creating graphs. Continuous variables are presented either as means + SD or medians and interquartile ranges. Data were tested for normal distribution applying Shapiro-Wilk test and for equality of variances by Brown-Forsythe test. Differences in variables between two groups were assessed by unpaired two-tailed Student’s *t*-tests. Differences in variables between more than two groups were assessed by Welch’s test followed by Dunnette’s T3 test for selected pairs of columns for normally distributed data with unequal variances, and by Kruskal Wallis tests followed by Dunn’s test for selected pairs of columns for not normally distributed data. Spearman rank correlations were calculated to assess relations between variables. Non-parametric partial correlations were calculated using SPSS Statistics Version 29 . p < 0.05 was considered to be statistically significant. *, p < 0.05; **, p < 0.01; ***, p < 0.001; ****, p < 0.0001.

## 3 Results

In this study, we investigated the putative immune stimulatory effects of commercial research grade albumins and therapeutic albumin, and compared these effects to immune stimulation enhanced by purified albumin from EDTA plasma of healthy donors. All albumins were characterized concerning their redox state in terms of Cys-34, as well as their non-esterified fatty acids (FA) and glycation (GSP) content per mole albumin. Furthermore, putative endotoxin contaminations were assessed. All albumins investigated are listed in Table 1.

**Table 1:** Albumin products investigated in this study.

**Terminus**	**Albumin product**	**Distributor**	**FA/ALB mol/mol**	**GSP/ALB (mol/mol)**	**HMA %**	**HNA1 %**	**HNA2 %**	**Endotoxin pg/mg**
C1	Plasma-derived albumin of 4 healthy donors	Purified at Med Uni Graz	0.5	0.4	80.9	16.9	2.3	16.7
C2	Plasma-derived albumin of 4 healthy donors	Purified at Med Uni Graz	0.4	0.5	76.4	21.5	2.0	2.6
Infusion	Therapeutic grade albumin infusion solution, N-Acetyltrypto-phane-deprivated	Behring	6.7	0.2	30.9	46.1	23.0	28.8
HSA	Albumin from human serum, A1653 (Lot SLBX4252)	Sigma Aldrich	1.1	0.7	28.2	53.2	18.6	21.6
- FA1	Fatty acid free albumin, A1887 (Lot SLCL2530)	Sigma Aldrich	0.1	0.9	41.0	46.2	12.8	47.0
- FA2	Fatty acid free albumin, A1887 (Lot SLBM7779V)	Sigma Aldrich	n.a.	n.a.	26.5	49.8	23.7	260.4
- FA3	Fatty acid free, globulin free albumin, A3782 (Lot SLCH3002)	Sigma Aldrich	0.1	0.4	42.9	46.3	10.8	151.8
Glyc	Glycated albumin, A8301 (Lot SLCG2829)	Sigma Aldrich	4.3	2.5	26.4	42.9	30.7	731.2
Rec1	Recombinant Albumin (from rice), A9731 (Lot SLCJ1701)	Sigma Aldrich	4.9	0.2	35.0	55.0	10.0	47.0
Rec2	oHSA, recombinant HSA (from rice)	Oryzogen	5.3	0.4	59.1	17.1	23.8	15.7

**Fig. 1:**
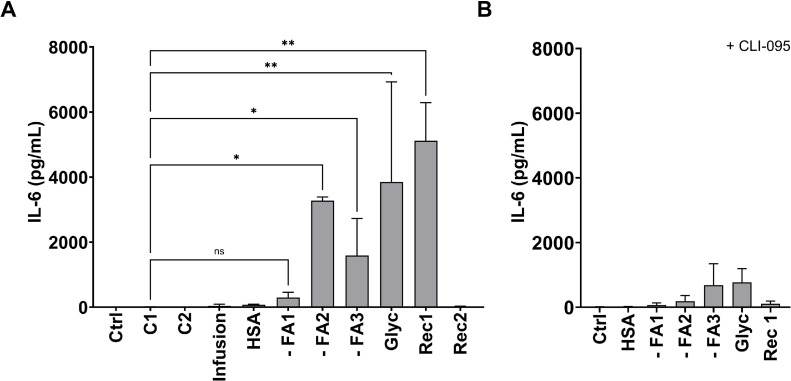
Incubation with different albumins stimulates IL-6 release by human PBMC. Freshly isolated PBMC of healthy donors were incubated in the absence (Ctrl) or presence of different albumins (1 mg/mL) for 4 h. IL-6 concentration in the supernatant was determined by ELISA. Data are presented as median + IQR. Each albumin was incubated with at least three different donor PBMC preparations. Statistical analysis was performed applying Kruskal-Wallis test followed by Dunn’s test for selected pairs of columns (compared to C1). *, p < 0.05; **, p < 0.01; ns, not significant. (B) Cells were incubated in the presence of albumins and TLR4 inhibitor CLI-095. Data are presented as mean + SD of 2 independent experiments.

**Fig. 2:**
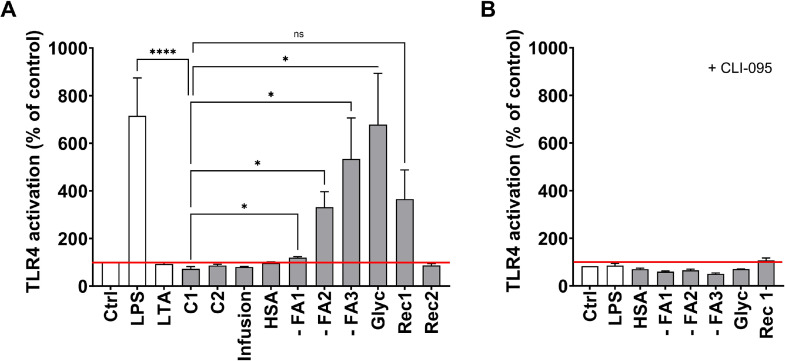
TLR4 is activated by incubation with different albumins. HEK-Blue™ TLR4 cells were incubated in the presence of different albumins (1 mg/mL). LPS was added as positive control for TLR4 activation. As negative control, cells were incubated in the absence of albumin (Ctrl). (A) Results are presented as means + SD of at least 3 independent incubations. *, p < 0.05; ****, *p* < 0.0001; ns, not significant by Welch’s test followed by Dunnette’s T3 test for selected pairs of columns (compared to C1). (B) Cells were incubated in the presence of CLI-095. Results are represented as means + SD of 2 independent incubations.

To investigate the immune stimulatory potential of albumin products, we incubated freshly isolated human PBMC with all albumins listed in Table 1 and measured the release of IL-6 into the supernatant as an inflammation marker. Incubation with - FA2, - FA3, Glyc, and Rec1 induced drastic release of IL-6 compared to albumin from healthy donors (C1), which led to the release of 4 (2.1 – 15) pg/mL IL-6 (Fig. 1A). With a release of 297 (199 – 458) pg/mL of IL-6, cells incubated with - FA1 also released considerable amounts of IL-6 into the supernatant. However, this difference to C1 was not statistically significant (p = 0.20). One of the reported key pattern recognition receptors involved in inflammation in PBMC is TLR4. Since co-incubation of PBMC with the TLR4-selective inhibitor CLI-095 significantily decreased cellular IL-6 release (Fig. 1B) it was assumed that pro-inflammatory responses elicited via albumin incubation were due to TLR4 activation. Due to these findings, we determined TLR4 activation by respective albumins using HEK-Blue™ TLR4 reporter cells. - FA1, - FA2, and - FA3 as well as Glyc led to a significant activation of TLR4 in HEK-Blue™ cells and Rec1 led to TLR4 activation close to significance level (p = 0.065) (Fig. 2A). The activation of TLR4 was completely prevented upon co-incubation with CLI-095 (Fig. 2B). Albumin induced IL-6 release by PBMC was strongly correlated with the activation of TLR4 in HEK-Blue™ TLR4 cells (r = 0.87, p = 0.002).

**Fig. 3:**
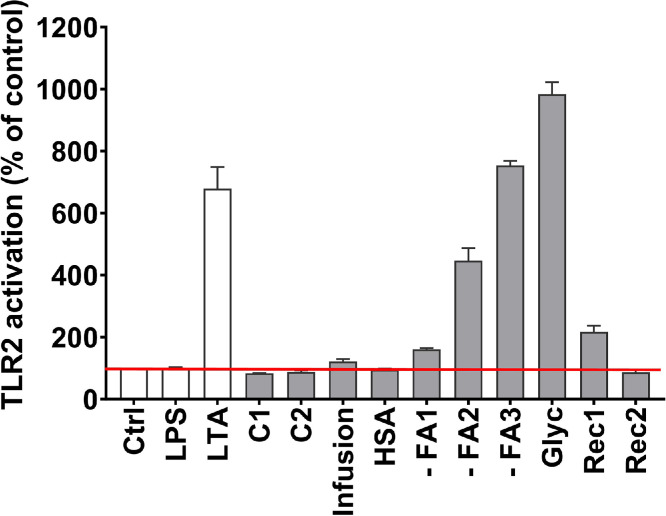
TLR2 is activated by different albumins. HEK-Blue™ TLR2 cells were incubated in the presence of different albumins (1 mg/mL). LTA was added as positive control for TLR2 activation. As negative control, cells were incubated in the absence of albumin (Ctrl). Results represent mean + SD of 2 independent experiments.

As shown in Fig. 1B, there is a residual TLR4-independent induction of IL-6 release by PBMC incubated with - FA1, - FA2, - FA3, Glyc, and Rec1. This may be, at least partly linked to an activation of TLR2, as shown by the activation of HEK-Blue™ TLR2 cells by these same research grade albumins (Fig. 3). Furthermore, IL-6 release by PBMC and TLR2 activation in HEK-Blue™ TLR2 cells were strongly correlated (r = 0.94; p = 0.0002). Additionally, a strong correlation between the activation of TLR4 and TLR2 by the albumin products was detected (r = 0.90, *p* = 0.0008) (Fig. 2, Fig. 3).

Many of the investigated parameters listed in Table 1 did not exhibit any significant correlations with the release of IL-6 by PBMC, neither the FA content (r = −0.27, p = 0.45) or the degree of glycation (GSP/ALB) (r = 0.26, p = 0.50), nor the albumin redox fraction HNA2 (r = 0.31, *p* = 0.39). HNA1 narrowly failed to reach significance (r = 0.64, p = 0.054). Only the HMA fraction showed a negative correlation with the IL-6 release (r = -0.77, p = 0.01). Importantly, some of the commercial albumins tested contained considerable amounts of endotoxin which was strongly correlated to IL-6 release by PBMC (r = 0.93, p = 0.0003). Similarly, the activation of TLR4 was strongly correlated only with the endotoxin content (r = 0.82, p = 0.005), but not with the FA content (r = −0.20, p = 0.61) or GSP/ALB (r = 0.38, p = 0.31). Additionally, there were no correlations of TLR4 activation with any albumin redox fraction (HMA: r = −0.58, p = 0.09; HNA1: r = 0.55, p = 0.10; HNA2: r = 0.35, p = 0.33).

Also, TLR-2 activation showed no correlation with the FA content (r = −0.10, p = 0.81) or GSP/ALB (r = 0.20, p = 0.60) nor with the albumin redox fractions HNA1 (r = 0.55, p = 0.10) and HNA2 (r = 0.37, p = 0.30). We found a significant negative correlation of HMA with the activation of TLR2 (*r* = −0.70, p = 0.03). Although endotoxin is not a ligand for TLR2, we detected a very strong correlation of the endotoxin content with TLR2 activation (r = 0.94, p = 0.0002) suggesting that the presence of endotoxin in these albumin products may correlate with other microbial components that are able to directly stimulate TLR2.

Since the albumin redox fraction HMA turned out to be significantly negatively correlated with the endotoxin content (r = −0.73, p = 0.02), we calculated non-parametric partial correlations between the albumin redox fractions and IL-6 release and TLR4/2 activation with endotoxin content as control variable. This resulted in a loss of the bivariate correlation between HMA and IL-6 (r = −0.36, p = 0.34) as well as with TLR2 activation. All bivariate correlations with the albumin redox state are shown in Table 2, non-parametric partial correlations with endotoxin as control variable are listed in Table 3.

**Table 2:** Spearman rank correlations of albumin redox state with TLR4 and TLR2 activation in HEK-Blue™ TLR cells, IL-6 release by PBMC, and endotoxin content.

		**HMA**	**HNA1**	**HNA2**
TLR4 activation	r	−0.58	0.55	0.35
	*P*	0.09	0.10	0.33
TLR2 activation	r	−0.70	0.55	0.37
	*P*	**0.03**	0.10	0.30
IL-6 release	r	−0.77	0.64	0.31
	*P*	**0.01**	0.05	0.39
Endotoxin content	r	−0.73	0.49	0.46
	*P*	**0.02**	0.15	0.19
	n	10	10	10

**Table 3:** Non-parametric partial correlations of albumin redox state with TLR4 and TLR2 activation in HEK-Blue™ TLR cells and IL-6 release by PBMC using endotoxin content as control variable.

		**HMA**	**HNA1**	**HNA2**
TLR4 activation	r	0.06	0.30	−0.06
	*P*	0.88	0.44	0.88
TLR2 activation	r	−0.06	0.30	−0.18
	*P*	0.88	0.44	0.64
IL-6 release	r	−0.36	0.56	−0.35
	*P*	0.34	0.12	0.35

**Fig. 4:**
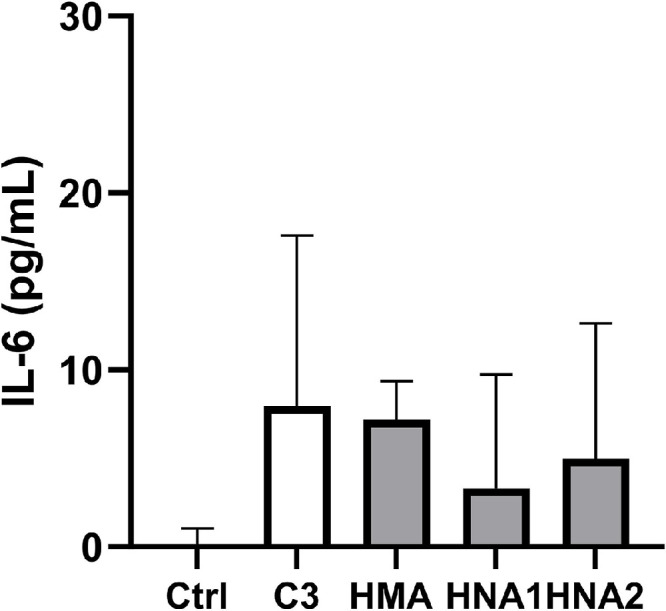
Albumin redox state does not affect IL-6 release by PBMC. Albumin (C3) and thereof prepared HMA, HNA1, and HNA2 from 3 different donors were incubated with separately isolated human PBMC for 4 h at standard conditions. As negative control, cells were incubated in the absence of albumin (Ctrl). IL-6 concentration in cell culture supernatants was determined by ELISA. Data are shown as median + IQR of 9 incubations in total.

To investigate the direct effect of the albumin redox state on IL-6 release by PBMC we separately incubated the cells with albumin of three healthy donors (C3) and thereof prepared albumin redox fractions. The endotoxin content in all these preparations was low (HSA: 12.9 ± 8.7 pg/mg; HMA: 11.6 ± 8.2 pg/mg; HNA1: 15.2 ± 10.4 pg/mg; HNA2: 8.7 ± 6.5 pg/mg). As shown in Fig. 4, IL-6 release in PBMC was not induced by incubation with reduced or oxidized albumin fractions, respectively.

**Fig. 5:**
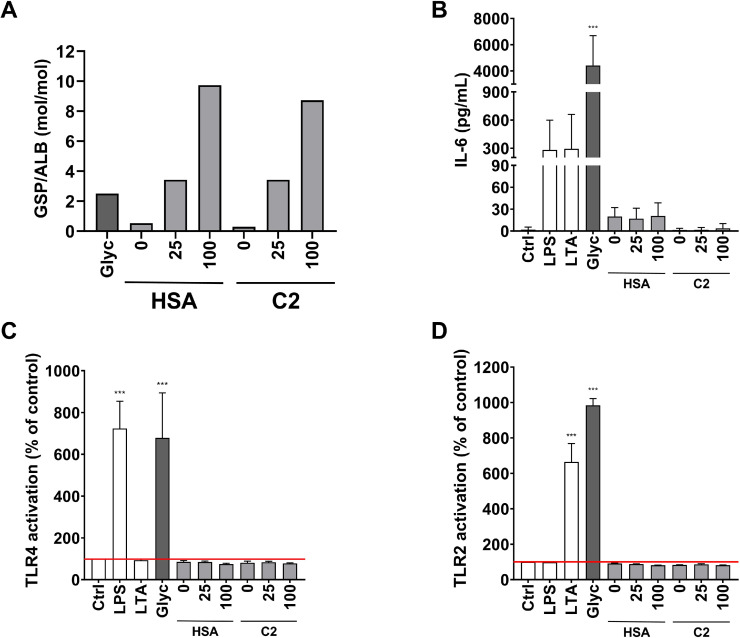
Effect of albumin glycation on IL-6 release by PBMC and TLR4 and TLR2 activation in HEK-Blue™ TLR cells. Research grade albumin (HSA) and healthy control albumin (C2) were incubated in the absence or presence of 0, 25, or 100 mmol/L glucose for 3 weeks at 37 °C and their potential to stimulate IL-6 release or to activate TLR4 and TLR2 was compared to commercial glycated albumin (Glyc). (A) Degree of glycation of albumins. (B) IL-6 release by human PBMC. (C) TLR4 and (D) TLR2 activation in HEK-Blue™ TLR cells. As negative control, cells were incubated in the absence of albumin (Ctrl). As positive controls, cells were incubated with LPS for TLR4 activation and LTA for TLR2 activation, respectively. Statistical analysis was performed applying one-way ANOVA followed by Dunnette’s test for multiple comparisons (compared to Ctrl).

It is reported that glycated albumin stimulates the expression of pro-inflammatory cytokines in different cell types [7,14,15]. In our experiments, incubation with a commercial glycated albumin (Glyc) also led to the release of IL-6 by PBMC as well as to an activation of TLR4 and TLR2 in the respective HEK-Blue™ TLR reporter cells (Fig. 1, 2, 3). However, as already mentioned above, there was no correlation of GSP/ALB of all albumins tested with the IL-6 release and the activation of TLR4 or TLR2. Importantly, Glyc contained 731.2 pg/mg endotoxin, the highest amount of all commercial albumins tested within this study. To determine the immune stimulatory effect of glycated albumin without confounding endotoxin we glycated commercial albumin with low endotoxin content (HSA, 21.6 pg/mg endotoxin) as well as healthy donor albumin (C2, 2.6 pg/mg endotoxin) in vitro in the presence of 25 mM or 100 mM glucose under sterile conditions. The endotoxin content did not change during this procedure (data not shown). Incubation with 25 mM glucose resulted in a glycation grade of 3.4 mol/mol which was similar to the commercial product Glyc (2.5 mol/mol), the degree of glycation after incubation with 100 mM glucose was considerably higher (8.7 and 9.7 mol/mol in HSA and C2, respectively). However, none of the in vitro glycated albumins caused an elevation of IL-6 release by PBMC nor an activation of TLR4 or TLR2, respectively, in HEK-Blue™ TLR cells (Fig. 5B, C, D).

**Fig. 6:**
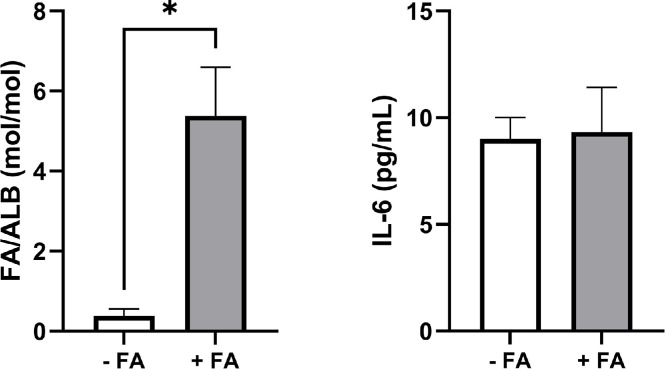
Ex vivo fatty acid loading does not affect IL-6 release by PBMC. Donor albumins (C3) were incubated in the presence of lipolysis-stimulated murine fat pads and thereby loaded with FA (left panel). Freshly isolated human PBMC were incubated with FA laden albumins for 4 h and IL-6 release was determined by ELISA (right panel). Statistical analysis was performed applying paired Student’s *t*-test. Data are presented as mean + SD, n = 3 different donor albumins (C3). *, p < 0.05.

Although we now know that many research grade albumins contain endotoxin contaminants, we have also identified two commercial albumins that were highly loaded with FA and led to increased IL-6 release by PBMC (Glyc and Rec1). To investigate the potential pro-inflammatory effects of massive FA loading of albumin we loaded three different donor albumins (C3) containing low endotoxin concentrations (12.9 ± 8.7 pg/mg) with FA by incubation with forskolin-stimulated murine fat pads. Although a high loading of 5.4 ± 1.2 mol FA per mole albumin was achieved, this treatment did not affect IL-6 release by PBMC (Fig. 6).

## 4 Discussion

In this study, we were able to show that various human serum albumin preparations, ranging from therapeutic albumin to serum derived or recombinant research grade albumins, exhibit pro-inflammatory potential to markedly different degrees. The investigated albumins differed greatly in terms of their FA content, degree of glycation, and their redox state. Each of these properties has been previously associated with pro-inflammatory effects in various studies. Importantly, many albumin products tested in this study contained significant amounts of endotoxin. While the latter was, unsurprisingly, strongly associated with the release of IL-6 by PBMC, we could not find any correlations of IL-6 release with the FA content or glycation. Correlations with the albumin redox state also indicated to be only a covariate of the endotoxin content. We found an activation of TLR4 and TLR2 by different research grade albumins, including FA free, glycated and recombinant albumin, which was strongly correlated to IL-6 release by PBMC as well as the endotoxin content of the products.

Interestingly, at first glance our data suggested strong negative bivariate correlations of HMA fraction and IL-6 release, as well as TLR4 and TLR2 activation. Conversely, this means that the release of IL-6 and the activation of TLR are associated with increased HNA fractions, although there was no clear direction towards HNA1 or HNA2. However, these correlations were lost when correcting for endotoxin levels in partial correlation analysis suggesting that the observed pro-inflammatory association was confounded by higher endotoxin content and maybe by additional contaminants in more oxidized albumin preparations. Magzal et al. reported that incubation of endothelial cells with albumin from hemodialysis patients led to slightly increased pro-inflammatory cytokine expression, a response that was impaired by treatment with dithiothreitol (DTT) [10]. Whether this effect was due to the albumin redox state in terms of Cys-34 itself or other DTT induced mechanisms such as the reduction of intramolecular disulfide bonds and/or conformational changes remains to be elucidated. In our experiments, the pro-inflammatory potential of in vitro produced HNA1 was very low and the IL-6 release by PBMC was essentially the same in cells incubated with HNA1 or HMA, which was prepared by incubation with N-acetylcysteine, a milder reducing agent than DTT. Thus, our data do not point towards an inflammatory potential of HNA1. However, since cultured cells are known to rapidly create a reducing environment for themselves at standard conditions a putative pro-inflammatory effect of HNA1 could have been prevented by cellular reduction to HMA in our experimental setting [16]. Furthermore, the situation in vitro does not necessarily correspond to the situation in vivo. Therapeutic albumin infusion solutions usually contain very high levels of oxidized albumin fractions. Despite that, their application is capable of reducing inflammation in patients with acutely decompensated cirrhosis which actually gives reason to believe that there are no adverse in vivo effects of HNA1 in patients [17].

It has been described that albumin from diabetic patients may have pro-inflammatory properties, probably due to covalent modification with glucose forming fructosamines or advanced glycation endproducts (AGEs) [9,18]). To address this question, several groups tested either commercial or in vitro glycated albumins for their pro-inflammatory potential [7,14,19]. The methods for glycation varied regarding the used albumin products, glycation reagents (e.g. glucose, methylglyoxal (MGO), glycoaldehyde, etc.) and incubation times [9,18,20]. In the present study, we performed the glycation by incubating albumin with d-glucose for 3 weeks at 37 °C, reflecting rather physiological conditions. According to the manufacturer’s description, the commercial albumin Glyc had also been glycated with glucose. We measured the GSP content in terms of fructosamines in all albumins used, however, we did not analyze AGEs such as carboxymethyllysine or MGO-derivatives, which have been implicated in inflammatory signaling [[21], [22], [23]]. Likely, there might be variations in AGEs among these products. The estimation of the pro-inflammatory effects of AGE-modified albumin in studies using commercial albumins may be confounded by the high endotoxin content of some products, which likely overrides the true pro-inflammatory impact of AGEs.

The magnitude of TLR activation by (saturated) FA is controversially discussed and ranges from clearly recognizable to even not existing [[24], [25], [26], [27]]. In our study, we measured albumin products within a broad range of molar ratios of FA to albumin from 0.1 to 6.7 mol/mol. The FA content was especially high in Glyc, Rec1, Rec2 as well as in Infusion. The latter contains the medium-chain FA caprylic acid as stabilizer, FA load of the other products may be of different composition. We found no correlations of FA content neither with TLR4/2 activation nor with IL-6 release. Additionally, we loaded albumin to a high extent ex vivo with FA from murine adipose tissue, but this did not induce IL-6 release by PBMC. Especially saturated FA are described to activate TLRs [28] and our lipid laden albumins certainly contained some of them, as lipolysis stimulated murine fat pads, as used in the present study, should release both, saturated and unsaturated FA from their triglyceride depots. This indicates that there is no FA mediated activation of TLRs. However, unlike endotoxin which activates inflammatory signaling within minutes, TLR activation by FA may take longer exposure times of several hours [29,30]. The 4 h incubation time of PBMC may thus have been insufficient in detecting FA-induced TLR activation. Further studies are necessary to shed light on this topic.

It is important to mention that albumin is not a uniform entity. Products vary in their origin, purification method, ligand content, etc. The subsequent delipidation or glycation of purified albumin requires further treatment enhancing the risk of bringing in contaminants. It is essential for patient safety that therapeutic albumin is free of microbial contaminations. Therefore these products are tested to be pyrogen-free and we did not see any immuno stimulating effects in our experiments either. On the other hand, the information about putative contaminants in research grade albumins is often not provided. In most cases, PTMs and potential ligands remain unidentified, too. Although such variations may be acceptable in some research applications, these may seriously compromise studies like investigating the inflammatory role of albumin.

One weakness of our study lies in the inter-donor variability of the pro-inflammatory responses of PBMCs. All albumins were incubated with at least three separate preparations of donor PBMC resulting in substantially varying IL-6 concentrations in the media. However, in every experiment, IL-6 release by cells incubated with - FA2, - FA3, Glyc, and Rec1 clearly stood out from the incubations with the other albumins suggesting the robustness of our core findings. We did not investigate the whole range of possible signal cascades for inflammatory responses; this would have been far beyond the scope of this study. However, we could identify pitfalls in the interpretation of albumin-related research and where special attention is essential.

From our observations, we propose that albumin products should be systematically characterized prior to their use in the respective experiments, with specific attention to masked endotoxin. Differences in these products should be strictly considered as their underlying causes may influence experimental outcomes, particularly in inflammation-related research. To ensure accurate detection, unmasking steps such as proteinase K digestion [31], the addition of heparin [32] or bivalent cations [11] should precede endotoxin testing. Where endotoxin masking is suspected, HEK-Blue™ TLR4 reporter cell assays may offer a more reliable alternative to the LAL assay [33]. The additional use of other HEK-Blue™ TLR reporter cells further enables additional quality assurance for impurities that are not detected in the LAL assay.

In conclusion, data from our study underline the variability and complexity of albumin products and emphasize the importance of critical product characterization in research. The potential of albumin’s modifications, ligands, or contaminants to influence experimental outcomes must be taken seriously to avoid misinterpretation and to ensure reproducibility of results

## Funding

This work was supported by the City of Graz (A 16–46033/2019).

## Declaration of generative AI and AI-assisted technologies in the writing process

During the preparation of this work the authors used ChatGPT-4 in order to improve readability and language of the manuscript. After using this tool, the authors reviewed and edited the content as needed and take full responsibility for the content of the present article.

## CRediT authorship contribution statement

**Margret Paar:** Writing – original draft, Visualization, Funding acquisition, Formal analysis, Conceptualization. **Vera H. Fengler:** Methodology, Formal analysis. **Martina Schweiger:** Methodology. **Christine Rossmann:** Writing – review & editing, Methodology. **Christoph Nusshold:** Writing – review & editing, Methodology. **Martina Mairold:** Methodology. **Doris Payerl:** Methodology. **Gerhard Cvirn:** Writing – review & editing, Methodology. **Karl Oettl:** Writing – review & editing, Formal analysis, Conceptualization.

## Declaration of competing interest

The authors declare the following financial interests/personal relationships which may be considered as potential competing interests:

Margret Paar reports financial support was provided by City of Graz. Karl Oettl reports a relationship with Takeda Pharmaceuticals International AG that includes: consulting or advisory. If there are other authors, they declare that they have no known competing financial interests or personal relationships that could have appeared to influence the work reported in this paper.

## Data Availability

Data will be made available on request.
